# Refractory and Recurrent Skin Manifestations in an Adult With Selective Immunoglobulin M Deficiency

**DOI:** 10.7759/cureus.59015

**Published:** 2024-04-25

**Authors:** Hayakazu Sumida, Shinichi Sato

**Affiliations:** 1 Department of Dermatology, Graduate School of Medicine, The University of Tokyo, Tokyo, JPN; 2 Scleroderma Center, The University of Tokyo Hospital, Tokyo, JPN; 3 SLE Center, The University of Tokyo Hospital, Tokyo, JPN

**Keywords:** eosinophil, ige, skin, igm deficiency, immunoglobulin m

## Abstract

Selective immunoglobulin M (IgM) deficiency (sIgMD) is a rare immunodeficiency disorder characterized by decreased serum levels of IgM. Symptoms of sIgMD include repeated infections and allergic manifestations such as asthma and allergic rhinitis. The etiology and pathology of sIgMD remain largely unknown. Moreover, no genetic cause of sIgMD and associated symptoms has been established. Herein, we describe a 47-year-old female with sIgMD who presented with repeated fevers of unknown cause since childhood. She was referred to our department because of recently developed severe dermatitis without a history of atopic dermatitis or asthma. In addition to histological evaluation by skin biopsy, immunological parameters were investigated in her peripheral blood, and the cellular immunity profile was determined by flow cytometry. The patient with refractory skin manifestations was found to have sIgMD with normal surface levels of IgM in the B cells. Along with recurrence and exacerbation in dermatitis, she showed an increase in peripheral blood eosinophils and serum IgE levels, suggesting an underlying allergic mechanism. The present case strongly indicates the importance of measuring serum IgM levels when seeing patients with recurring fever and intractable skin manifestations.

## Introduction

Selective IgM deficiency (sIgMD) is a form of dysgammaglobulinemia classified under primary humoral immunodeficiencies and is listed in the International Union of Immunological Societies (IUIS) classification of inborn errors of immunity (IEI) [1]. sIgMD is diagnosed when the serum IgM is two standard deviations (2 SD) below the mean, accompanied by normal IgG, IgA, and T cell numbers/functions according to the European Society for Immunodeficiencies (ESID) criteria. However, the definition of sIgMD is still controversial, and some express that sIgMD should be defined without the IgG subclass abnormalities so that we can understand various phenotypes underlying various symptoms associated with IgM deficiency [2]. Based on these opinions, a recent update to the Classification in the IUIS Expert Committee describes the main criteria as the absence/reduction in serum IgM without additional features [3].

The etiology and pathophysiology of sIgMD are still unknown, and the underlying genetic or molecular mechanism has remained elusive. Given that most patients show normal numbers of circulating surface IgM-positive B cells, the pathogenesis of sIgMD cannot be explained simply by defects in IgM [4]. With normal surface IgM expression in B cells, some studies revealed that insufficient synthesis of secreted IgM mRNA caused the inability of B cells to differentiate into IgM-secreting cells [5, 6].

Almost half of the sIgMD patients were reported to have autoimmune diseases, including Hashimoto’s thyroiditis and systemic lupus erythematosus (SLE) [7]. Moreover, patients with sIgMD may present with allergic manifestations such as allergic rhinitis and asthma. Regarding skin manifestations, a multicenter study of pediatric sIgMD patients showed that some patients presented with skin symptoms, including urticaria [8]. However, there are few reports regarding skin symptoms in sIgMD patients, not only in children but especially in adults.

## Case presentation

**Figure 1:**
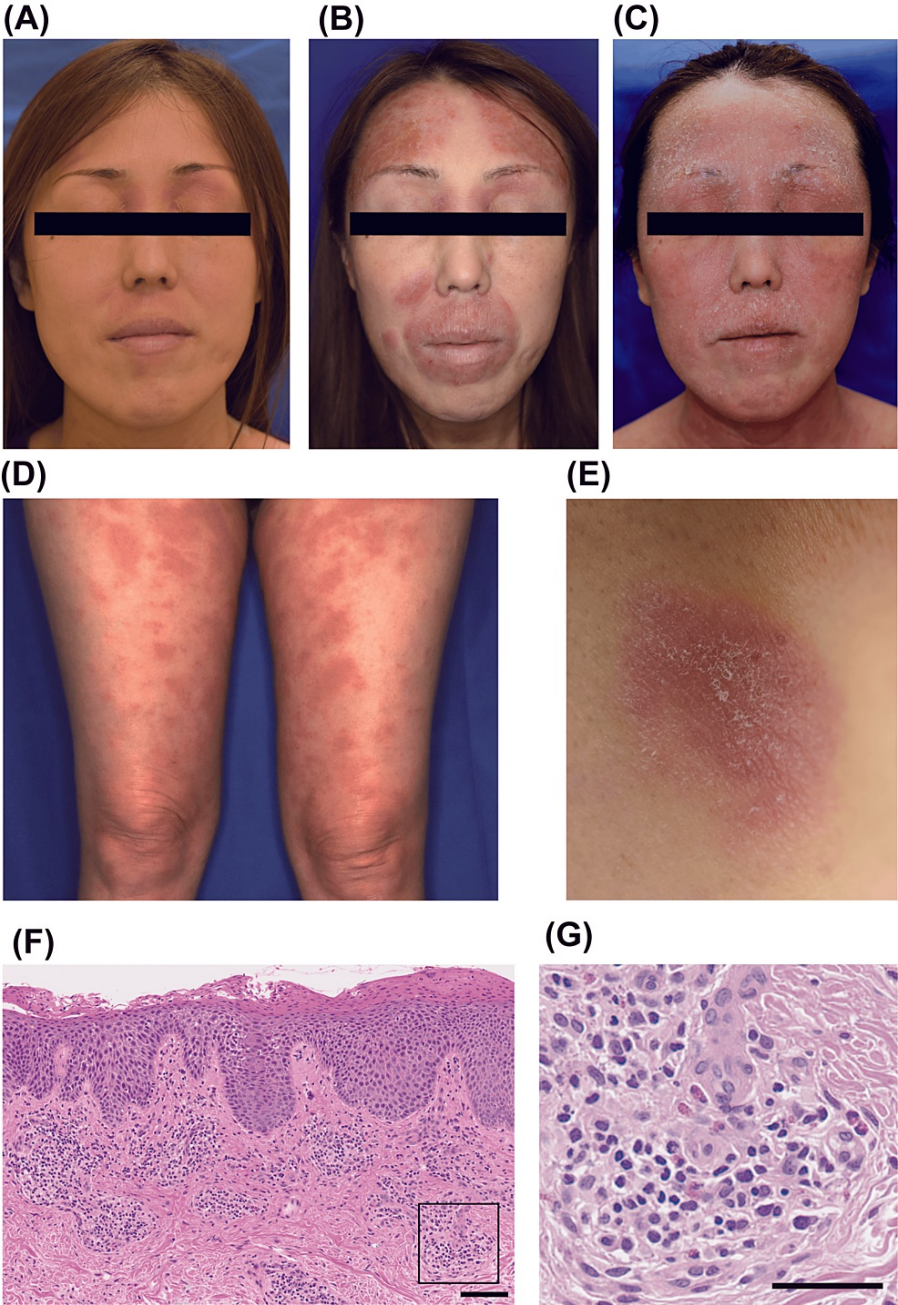
Recurrent and refractory dermatitis. (A-C) Skin manifestations on the face under stable (A), deteriorating (B), and most severe (C) conditions. Skin lesions over the lower limbs (D) and indurated and well-circumscribed erythema on the shoulder (E). Hematoxylin and eosin staining pictures from the lesion on the shoulder (F-G). (F) Superficial perivascular inflammatory infiltrate in the dermis. The epidermis showed irregularly elongated rete ridges with parakeratosis on the surface layer. Scale bar, 100 μm. (G) High-magnification view of the black-lined square in (F). Infiltrating cells included not only lymphocytes but also eosinophils. Scale bar, 50 μm.

Here, we present a 47-year-old Japanese woman whose main complaint was recurrent eczema scattered throughout the body. She had been suffering from repeated fevers of unknown cause regardless of skin symptoms since she was approximately 10 years old. In terms of skin manifestations, she had suffered from recurrent eczematous lesions throughout the body, including her face and limbs, especially in the last few years. The patient was referred to our department for these refractory eczematous lesions. Physical examination revealed erythema with hyperkeratosis and induration on the face, trunk, and limbs (Figure 1A-1E). In addition to its distribution, skin induration with relatively clear boundaries is not typical for atopic dermatitis. Although these skin eruptions are accompanied by itching, especially at night, they do not disappear, suggesting that these skin rashes are eczematous changes but not urticaria. The histological analysis of skin biopsy from the back lesions found a superficial perivascular infiltrate of lymphocytes and eosinophils. Although lymphocytes also infiltrated into the epidermis, no findings were found that strongly indicated cutaneous T cell lymphoma (CTCL). The epidermis showed irregularly elongated rete ridges, with parakeratosis on the surface layer (Figure 1F-1G).

Representative data at the time of exacerbation of skin eruption are as follows. The total leucocyte count was 6,500 cells/mm^3^ with eosinophils (16.4%). Although the C-reactive protein level (0.30 mg/L) was within normal limits, there was elevated thymus and activation-regulated chemokine (TARC)/CCL17 (8,732 pg/mL). Quantitative immunoglobulins demonstrated an elevated IgE level of 2,237 IU/mL (reference range, 0-170 IU/mL), and her baseline IgE levels were around 1,000 IU/mL. Both serum IgA and IgG levels were in reference range values (238 mg/dL and 1,546 mg/dL, respectively). However, serum IgM was low, with a value of 28 mg/dL (reference range, 50-269 mg/dL). Following further repeated investigation for several years leading up to the present still showing low levels of IgM regardless of skin rash and other immune-related parameters, the patient was subsequently diagnosed with sIgMD.

We further conducted tests for complications of SLE and other autoimmune diseases. Antinuclear antibodies were negative and under no systemic immunomodulators, and no other serological or clinical findings suggestive of autoimmune disease were found. As a part of the investigation into the recurring fever, we also conducted genetic testing for autoinflammatory diseases such as familial Mediterranean fever, cryopyrin-associated periodic syndrome, tumor necrosis factor receptor-associated periodic syndrome, and hyper-IgD syndrome. However, no known related genetic abnormalities were found in the test results. Further consultation with a hematologist helped us rule out hematological malignancies, such as malignant lymphoma.

**Figure 2:**
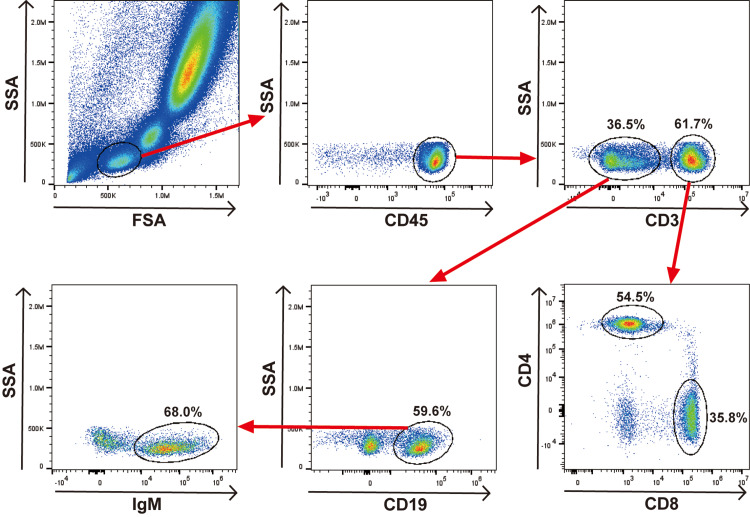
Flow cytometry analysis of peripheral blood in the patient. There were no abnormalities or deviations specifically in CD19^+^ B cell and CD3^+^ T cell fractions pregated on CD45^+^ cells. There was a normal percentage of B cells bearing IgM on their surface. Numbers show the percentage of cells in the indicated gate. Data were acquired using a CytoFLEX-S instrument (Beckman Coulter, Indianapolis, IN) and analyzed using FlowJo v10 software (FlowJo, LLC, Ashland, OR). SSA: side scatter area; FSA: forward scatter area; IgM: immunoglobulin M

Subsequently, immunological studies in her peripheral blood were conducted concerning low serum IgM levels, after obtaining written consent approved by the Research Ethics Committee of the Faculty of Medicine of The University of Tokyo. Flow cytometry analysis revealed no particular abnormalities or deviations specifically in CD19^+^ B cell and CD3^+^ T cell fractions pregated on CD45^+^ cells. Furthermore, the percentage of CD4^+^ T cells (54.5%), CD8^+^ T cells (35.8%), and the CD4/CD8 ratio (1.52) in CD3^+^ T cells were in reference ranges. In addition, further analysis of IgM surface expression in B cells revealed a comparable percentage of B cells bearing IgM on their surface (68%) to that of healthy donors, as shown in a previous report [9], which also showed no significant difference in B cell surface IgM expression between sIgMD patients and healthy donors (Figure 2).

Although skin manifestations were controlled to some extent with topical corticosteroids, both proactive and rank-down topical corticosteroid therapy could not prevent the recurrence of severe eczematous skin inflammation with increased serum IgE levels and peripheral blood eosinophils. Each recurrent skin rash needed potent or very potent topical corticosteroids without systemic corticosteroids to control the patient's skin symptoms.

## Discussion

Several medical studies have indicated that decreased serum IgM levels may be associated with recurrent infections such as respiratory tract infections, autoimmune diseases such as SLE, and several primary immunodeficiency types [10, 11]. A recent study of pediatric sIgMD reported that some patients showed skin symptoms such as urticaria [8]. However, it is unclear what kind of skin manifestation the disease is associated with. Here, we showed a sIgMD patient with recurrent severe dermatitis accompanied by elevated IgE and eosinophils. This case indicated the importance of considering the possibility of sIgMD when we see severe dermatitis that is thought to be caused by an allergic mechanism.

In terms of allergic manifestations, patients with sIgMD are sometimes associated with asthma and allergic rhinitis [11]. However, there are few descriptions of skin symptoms other than urticaria, and to our knowledge, severe recurrent eczematous skin symptoms such as this case have not been reported. In this case, IgE and eosinophil levels were elevated as her dermatitis was exacerbated, suggesting underlying allergic mechanisms, although she did not show asthma and allergic rhinitis. The skin eruptions appeared sporadically over a short period and were often accompanied by induration and/or clear borders, making it difficult for us to diagnose her with atopic dermatitis. Regarding autoimmune diseases, no significant results were obtained, although we conducted not only hematological screening for collagen diseases but also gene testing for autoinflammatory diseases. Furthermore, she did not have the clinical features of hyper-IgE syndrome such as characteristic facial appearance, joint hyper-extensibility, and scoliosis. These results imply that low serum IgM levels are associated with recurrent dermatitis.

In our patient, the shift toward a Th2-dominant immune response is suggested by her elevated serum levels of TARC, a Th2 chemokine. Patients with atopic dermatitis are well known to have elevated IgE and TARC levels in their serum, and these levels usually correlate with the severity of the disease [12]. However, dermatitis in this case is not typical of atopic dermatitis as described above. The mechanism through which low serum IgM levels cause a Th2 response shift remains unclear. Moreover, the ESID Registry defines patients with sIgMD as patients whose serum IgM levels are repeatedly low and whose serum IgA, total IgG, and IgG subclass levels are normal. In contrast, there is no definition for IgE, and high serum IgE levels were observed in some cases similar to this case [13]. Further investigation is needed to examine the possibility that low IgM levels directly or indirectly affect the control of B cell class switching to IgE.

## Conclusions

This case describes a patient with recurrent fever from childhood and severe dermatitis noticeable from adulthood. Her laboratory findings implicated an underlying allergic mechanism and led to the diagnosis of sIgM. This case report strongly indicates the importance of measuring serum IgM levels while keeping in mind the possibility of sIgMD when we see patients with Th2-type eczema that is refractory to conventional treatments.
